# Ocular Implications of COVID-19 Infection and Vaccine-Related Adverse Events

**DOI:** 10.3390/jpm14080780

**Published:** 2024-07-23

**Authors:** Uday Pratap Singh Parmar, Pier Luigi Surico, Rohan Bir Singh, Mutali Musa, Anna Scarabosio, Giorgio Surico, Antonino Maniaci, Salvatore Lavalle, Fabiana D’Esposito, Antonio Longo, Andrea Russo, Caterina Gagliano, Marco Zeppieri

**Affiliations:** 1 Department of Ophthalmology, Government Medical College and Hospital, Chandigarh 160047, India; upsparmar3112@gmail.com; 2 Schepens Eye Research Institute of Mass Eye and Ear, Harvard Medical School, Boston, MA 02114, USA; 3 Department of Ophthalmology, Campus Bio-Medico University, 00128 Rome, Italy; 4 Department of Optometry, University of Benin, Benin City 300238, Nigeria; 5 Department of Plastic Surgery, University Hospital of Udine, 33100 Udine, Italy; 6 Medicine and Surgery Department, University of Bari Aldo Moro, 70126 Bari, Italy; 7 Department of Medicine and Surgery, University of Enna “Kore”, Piazza dell’Università, 94100 Enna, Italy; 8 Imperial College Ophthalmic Research Group (ICORG) Unit, Imperial College, 153-173 Marylebone Rd., London NW1 5QH, UK; 9 Department of Neurosciences, Reproductive Sciences and Dentistry, University of Naples Federico II, Via Pansini 5, 80131 Napoli, Italy; 10 Faculty of Medicine, University of Catania, Piazza Università, 95123 Catania, Italy; 11 Eye Clinic Catania University San Marco Hospital, Viale Carlo Azeglio Ciampi, 95121 Catania, Italy; 12 Department of Ophthalmology, University Hospital of Udine, 33100 Udine, Italy

**Keywords:** ocular immunity, SARS-CoV-2, COVID-19, vaccination, side effects, ocular inflammation, adaptive immune response

## Abstract

The COVID-19 pandemic, caused by SARS-CoV-2, has significantly impacted various organ systems, including the eyes. Initially considered a primarily respiratory disease, it is now evident that COVID-19 can induce a range of ocular symptoms. Recognizing these ocular manifestations is crucial for eye care practitioners as they can serve as early indicators of the disease. This review consolidates current evidence on the ocular effects of COVID-19, identifying manifestations such as conjunctivitis, scleritis, uveitis, and retinopathy. The increasing prevalence of these symptoms highlights the importance of thorough eye examinations and detailed patient histories in COVID-19 cases. Potential routes of viral entry into ocular tissues and the underlying mechanisms, including direct infection, immune responses, and vascular involvement, are explored. Additionally, this review addresses ocular side effects associated with COVID-19 vaccines, such as corneal graft rejection, uveitis, and retinal issues. These findings emphasize the need for ongoing surveillance and research to ensure vaccine safety.

## 1. Introduction

Severe acute respiratory syndrome coronavirus (SARS-CoV-2) is a highly contagious and pathogenic virus that is known to cause a spectrum of human respiratory tract infections ranging from mild colds to severe respiratory distress syndrome. Coronavirus initially gained widespread attention following the 2003 outbreak of severe acute respiratory syndrome coronavirus (SARS-CoV) [1]. On 30 December 2019, Li Wenliang, an ophthalmologist at Wuhan Central Hospital, alerted fellow medical professionals about a potential outbreak resembling SARS. The World Health Organization (WHO) declared the Wuhan outbreak a Public Health Emergency of International Concern on 30 January 2020, and a pandemic on 11 March [2]. Since the onset of the pandemic, the WHO estimates that more than a billion confirmed cases of the coronavirus disease 2019 (COVID-19) and more than 6 million deaths worldwide have occurred [3].

Due to the virus’s potential to cause life-threatening respiratory issues, much of the research on coronaviruses (CoVs) center around the respiratory system. However, COVID-19 demonstrates a multifaceted nature, exhibiting neurotropism and endothelial tropism, alongside the capacity to provoke a systemic inflammatory response [4]. Consequently, a notable portion of COVID-19 patients may experience neurological and vascular symptoms. Manifestations of COVID-19 in the different organ systems should not be overlooked, as they could hint toward an alternative mode of transmission [4].

Ocular manifestations have been reported as potential initial indicators of COVID-19 [5]. Given the rapidly expanding body of literature on COVID-19, it is important to consolidate our current understanding of its ocular effects and discuss the implications for eye care practitioners. This article aims to review the existing evidence regarding the ocular involvement in COVID-19 and the potential underlying pathophysiological mechanisms. We additionally review the existing literature on the ocular complications associated with the COVID-19 vaccines currently in use.

## 2. Methods

This review utilized PubMed (https://pubmed.ncbi.nlm.nih.gov, accessed on 18 June 2024) and Reference Citation Analysis (RCA) (https://www.referencecitationanalysis.com, accessed on 18 June 2024). PubMed, a widely used and trusted biomedical literature database maintained by the National Library of Medicine (NLM), was selected as the primary resource for this research. Its extensive coverage of peer-reviewed journals in medicine and life sciences makes it an ideal tool for retrieving relevant scientific literature. A systematic approach was implemented to develop an effective search strategy. This process included identifying key concepts and terms related to the topic, including relevant medical terminologies, synonyms, and abbreviations. Key terms such as “COVID-19”, “SARS-CoV-2”, “vaccine”, “ocular”, “side effects”, “adverse events”, “eye”, “cornea”, “uvea”, “uveitis”, “retina”, and “retinopathy” were used. Boolean operators (AND, OR, NOT) were used to logically combine these terms, ensuring comprehensive coverage of pertinent literature while minimizing irrelevant results. The review search was updated to June 2024.

## 3. Ocular Involvement of COVID-19

### 3.1. Overview of SARS-CoV-2 Viruses

SARS-CoV-2 viruses are a family of positive sense, single-stranded ribonucleic acid (RNA), enveloped viruses. They belong to the Coronaviridae family within the suborder Coronavirineae and order Nidovirales [6]. The family is characterized by a spike (S) glycoprotein facilitating receptor binding and cell entry. Among the seven strains that are commonly reported to cause infection in humans, 229E, NL63, OC43 and HKU1 cause common colds, while Middle East respiratory syndrome (SARS)-CoV, severe acute respiratory syndrome (SARS)-CoV, and SARS-CoV-2 cause more severe respiratory infections [7].

### 3.2. Viral Transmission

According to the reported literature, COVID-19 transmission likely originated from bats, possibly reaching humans through other intermediate animals, potentially from the local sea food market in Wuhan [8]. Analysis shows a close similarity between the binding proteins Pangolin-CoV, Bat-nCoV and SARS-CoV-2, suggesting viral recombination [9]. Human-to-human transmission mainly occurs through respiratory droplets during coughing and sneezing or via fomites and viral contaminated surfaces [10]. Spike proteins are glycoproteins that protrude from the lipid envelopes of the viral particles and help with binding and entry into the cells. They do so by interacting with the angiotensin converting enzyme-2 (ACE2) receptors present on human cells facilitated by the serine protease TMPRSS2 [11].

### 3.3. Susceptibility to Ocular Involvement

There is evidence in the literature that the normal human conjunctiva harbors ACE2 receptors, implying the potential for SARS-CoV-2 to bind to the ocular surface [12]. Additionally, ACE1 and ACE2 enzyme expression has also been reported in the cornea and the aqueous humor, respectively [13]. Zhou L et al. reported a higher level of the receptor protein on the cornea and the limbus but reported lower levels in the conjunctiva [14]. Some degree of cross reactivity and binding might also be possible using the α2-3-linked sialic acid lectin binding sites, present in the epithelium of the nasolacrimal duct and lacrimal sac, which are used by adenoviruses and influenza viruses to gain entry into cells [15]. Notably, conjunctival swab samples from the tears of infected individuals have proven positive for SARS-CoV-2 RNA using the reverse-transcriptase polymerase chain reaction (RT-PCR). Other studies have reported the theoretical possibility of viral migration from the nasopharynx via the nasolacrimal duct. The hematogenous spread of the virus to the ocular tissues has also been studied [16]. Cerebral circulation is characterized by a relatively slower blood flow compared to other regions, which can create conditions that are more conducive to the entry and spread of pathogens, including viruses. This slower flow may allow viruses more time to interact with endothelial cells and potentially cross the blood–brain barrier. In the context of the olfactory bulb and cribriform plate, which are in close proximity to the nasal passages, this becomes particularly significant. The literature has explored the possibility of viral spread via the cerebral circulation, taking advantage of the slower blood flow and disseminating through the olfactory bulb and cribriform plate [17].

### 3.4. Prevalence and Incidence of Ocular Manifestations

Early data reported a lower prevalence of the ocular manifestations of COVID-19. Guan W et al. studied patient data from 552 hospitals with laboratory-confirmed COVID-19 in mainland China between December 2019 and January 2020. Among the 1099 patients included in the study cohort, only 9 (0.8%) were reported to have conjunctival congestion [18]. However, the recent literature has shown a greater prevalence of the ocular involvement of COVID-19. In the systematic review and meta-analysis reported by Nasiri N et al., the reported prevalence of ocular manifestations was estimated to be 11.03% in a study cohort of 8219 patients. They further reported the most common ocular manifestation to be dry eye or foreign body sensation (16%), followed by redness (13.3%), tearing (12.8%), itching (12.6%), eye pain (9.6%) and discharge (8.8%) [19].

### 3.5. Types of Ocular Manifestations

The studies on the different ocular manifestations of human coronavirus (CoV) infections are limited. However, CoVs have been reported to cause various ocular presentations in animals. Clinical conditions such as conjunctivitis, anterior uveitis, optic neuritis, and retinitis have been documented in feline and murine models [20]. In murine models, the virus is commonly reported to affect the posterior pole of the eye, characterized by choroiditis, retinal detachment, retinal degeneration, and retinal vasculitis [16]. The reported ocular manifestations of SARS-CoV-2 infection in humans are summarized in Table 1.

#### 3.5.1. Conjunctivitis

Conjunctivitis or excessive tearing has been reported as the initial or the sole symptom in some patients with COVID-19. Scalinci SZ et al. reported data on 5 patients having non-remitting conjunctivitis as the only presenting sign and symptom of COVID-19. These patients did not develop fever, malaise or any respiratory symptoms during the course of their infection and their diagnosis was confirmed using RT-PCR [21]. Navel V et al. reported a case of pseudomembranous and hemorrhagic conjunctivitis in a patient 19 days after onset of SARS-CoV-2 infection. Among children, some cases of COVID-19 have been closely linked with a presentation like that of Kawasaki disease, called multisystem inflammatory syndrome in children (MIS-C). The most common ocular manifestation in these patients was reported to be conjunctivitis [27].

#### 3.5.2. Scleritis and Episcleritis

Feizi S et al. reported two cases of anterior scleritis manifesting after the COVID-19 infection, the first one, in a 67-year-old woman that presented with necrotizing anterior scleritis one week after the onset of COVID-19, and the second one, in a 33-year-old man who presented with sectorial anterior scleritis 2 weeks after the onset of COVID-19. Workup identified no underlying autoimmune disease in any of these patients [22]. Otaif W et al. described the case of a 29-year-old man presenting with episcleritis as the first sign of an underlying COVID-19 infection [23]. Another study reported a case of nodular episcleritis in a patient with underlying COVID-19 infection [28].

#### 3.5.3. Uveitis

During the pandemic, an increase in the number of patients presenting with new-onset uveitis or recurrence of uveitis was noted. In a retrospective observational study conducted at the Beijing Tongren Hospital, 18 patients presented with symptoms of active uveitis within 4 weeks of their positive COVID-19 RT-PCR test [29]. Nine of these patients were reported to have new onset uveitis whereas the other nine had relapsed uveitis. Of the nine patients with new-onset uveitis, seven had a bilateral presentation. Among these patients, four had anterior uveitis, one had sympathetic ophthalmia, three patients had Vogt–Koyanagi–Harada (VKH) syndrome and one had multiple evanescent white dot syndrome (MEWDS) [29]. Patients presenting with various forms of posterior uveitis have also been reported in the literature [30].

#### 3.5.4. Retinopathy

The published literature has documented occurrences of both central retinal vein occlusions (CRVO) and central retinal artery occlusions (CRAO) in patients with underlying COVID-19 infection who do not exhibit the typical systemic vascular risk factors. Walinjkar et al. reported the case of a 17-year-old girl who presented with diminished vision 21 days after an underlying COVID-19 infection [24,25,31,32]. On examination, the patient had swelling of the optic disc, splinter hemorrhages, flame-shaped hemorrhages, and dot and blot hemorrhages. Cystoid macular edema and detachment of the neurosensory layer was noted on an optical coherence tomography examination, and a presumptive diagnosis of CRVO was made [24]. Gaba WH et.al. described a case of a 40-year-old man presenting with bilateral central retinal vein occlusion with severe coronavirus disease pneumonia [25]. Another study reported a case of impending CRVO in a patient with COVID-19. Fundoscopic and imaging examination in this patient revealed a fern-like hypo-autofluorescent appearance that is typical of ischemic CRVO (iCRVO) [32]. Heidarzadeh HR et al. reported a case of CRAO in a 44-year-old with a 1-week history of severe COVID-19 infection [31].

In another cross-sectional study, Invernizzi A et al. noted a direct correlation between retinal vein diameter with the severity of the underlying COVID-19 infection. The mean arterial diameter (MAD) and the mean venous diameter (MVD) were both noted to be significantly higher in COVID-19 patients (*p* < 0.0001). Tortuous veins were noted in 12.9% of the patient cohort [33]. Marinho et al. studied retinal and optical coherence tomography (OCT) changes in 12 patients with an underlying COVID-19 infection. All patients were reported to have hyper-reflective lesions at the level of the inner plexiform layer and ganglion cell layer. On fundus examination, four of these patients presented microhemorrhages and subtle cotton wool spots [34]. Similarly, Pereira LA et al. studied the ocular findings of 18 patients with confirmed severe COVID-19 and reported that 10 of these patients displayed abnormalities on dilated eye examinations. The common findings that were noted included flame-shaped hemorrhages, cotton wool spots and retinal sectorial pallor [35]. Soni A et al. reported two consecutive cases of acute retinal necrosis as a presenting manifestation in patients with a recent history of COVID-19. PCR from a vitreous sample was positive for Herpes simplex virus (HSV) in both patients. The proposed mechanism was that the immune dysregulation caused by the COVID-19 infection likely caused the reactivation of HSV, which together resulted in the presentation of retinal necrosis [36].

#### 3.5.5. Other Ocular Complications

Verkuli et al. described the case of a 14-year-old girl who presented with new onset abducens nerve palsy and papilledema and had hemorrhages on fundus examination of the optic disc. On lumbar puncture, the opening pressure was found to be 36 cm of H_2_O [37]. A diagnosis of pseudotumor cerebri syndrome associated with multisystem inflammatory syndrome was made. Other reported ocular complications include optic neuritis, papilledema, and palsy of the third and fourth cranial nerve [38,39,40]. Oscillopsia and central vestibular nystagmus have also been described as complications of an underlying COVID-19 infection [41,42]. 

## 4. Clinical Practice and Diagnostic Considerations

For accurate diagnosis of these ocular manifestations, it is of prime importance to take a detailed history regarding the onset and duration of the symptoms along with the accompanying characteristics. All patients should be questioned about any recent history of respiratory symptoms, fever, and travel history to assess the need for further evaluation of COVID-19. Repeated disinfection of equipment, including slit lamps and B-scan probes with 70% ethyl alcohol has shown to reduce coronavirus infectivity. Goldman tonometers can be sterilized with a 10% diluted sodium hypochlorite solution [43]. Ophthalmologists are also advised to prioritize the use of disposable equipment over reusable equipment. TonoSafe are disposable probes that can be used instead of reusable probes for tonometry.

## 5. Mechanisms of Ocular Infection by SARS-CoV-2

### 5.1. Entry Routes of the Virus into Ocular Tissues

**Figure 1:**
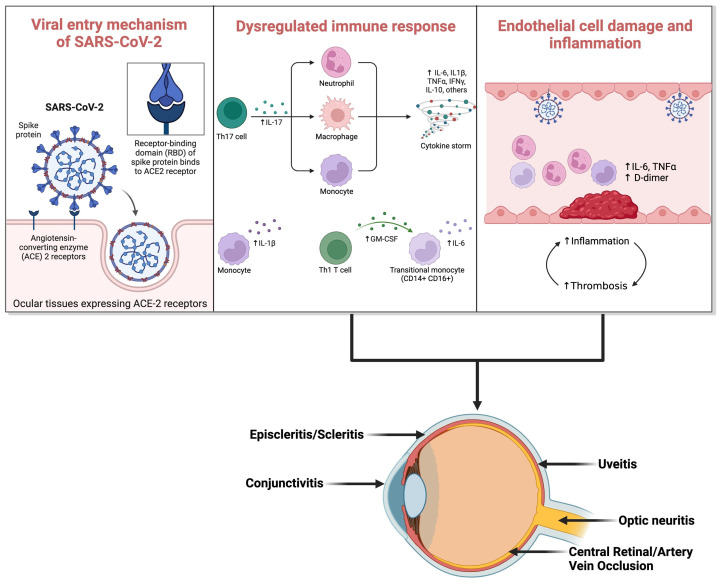
Viral entry mechanism of SARS-CoV-2 and subsequent immune response dysregulation leading to COVID-19 ocular manifestations. (ACE: Angiotensin-Converting Enzyme; IL-17: Interleukin-17; Th17: T-helper 17 cells; Th1: T-helper 1 cells; IL-6: Interleukin-6; IL-1β: Interleukin-1β; TNF-α: Tumor Necrosis Factor-α; IFN-γ: Interferon-γ; IL-10: Interleukin-10). (Created with Biorender.com).

Initially, the primary mode of viral entry into ocular tissues was thought to be direct inoculation onto the conjunctiva, binding to ACE2 receptors (Figure 1). However, this theory faced limitations due to the absence of activating serine protease in the conjunctiva [12]. Subsequent research has explored alternative pathways for viral transmission into ocular tissues. Literature reports indicate neuronal and hematogenous spread of the infection to the eyes [44]. Studies on the neuronal route suggest viral entry via retrograde transportation from the brain to the optic nerve [45]. Additionally, it has been suggested that following anterior segment inoculation, the virus can spread to other regions via corneal nerves connected to the trigeminal nerve [45,46]. Recent advancements underscore COVID-19 as a vascular disease, spreading hematogenously by infecting capillary endothelial cells. These cells express ACE2 and CD147 receptors, facilitating direct viral binding [47]. Another theory proposes virus transportation to the retina via leukocytes capable of crossing the blood–retinal barrier.

### 5.2. Underlying Pathophysiology and Immune Response in Ocular Tissues

The fundamental pathophysiological mechanisms involve viral infection triggering a proinflammatory and prothrombotic state [48]. This leads to the release of cytokines from proinflammatory cells, culminating in a cytokine storm and subsequent inflammatory conditions within the eye, such as conjunctivitis, uveitis, choroiditis, and retinitis. Notably, the cytokine storm induced by SARS-CoV-2 is marked by elevated levels of type 2 cytokines and diminished levels of type 1 cytokines. IL-33, a member of the IL-1 family secreted by epithelial cells, may further amplify this type 2 cytokine response [49]. Some sources liken the underlying pathophysiology to macrophage activation syndrome (MA.S.), particularly concerning the threshold model of SARS-CoV-2 infection [50]. Furthermore, certain studies suggest the involvement of the proptosis pathway and activation of the inflammasome in the inflammatory response [51].

The proposed experimental coronavirus retinopathy (ECOR) model suggests that retinal damage resulting from the viral infection unfolds in two distinct phases. Initially, immune cells infiltrate the retina, triggering the release of inflammatory mediators and instigating retinal inflammation. This phase is succeeded by viral clearance. Subsequently, the second phase ensues, characterized by the production of autoantibodies targeting the retina and pigment epithelium, which leads to photoreceptor and neuroretinal damage [52]. Moreover, in the context of ECOR, the observed retinal damage correlates with elevated levels of TNF-alpha and disrupted TNF signaling pathways [53].

When SARS-CoV-2 utilizes the ACE2 receptor to infiltrate local endothelial cells, it initiates endothelial damage and microangiopathic injury, thereby triggering localized inflammation. The byproducts of microvascular injury activate neutrophils, which, in turn, can harm the endothelial cell glycocalyx [54,55]. This endothelial disruption culminates in platelet activation and an overall state of hypercoagulability. COVID-19 associated coagulopathy (CAC) manifests as an endotheliopathy characterized by severe disturbances in D-dimer levels [56,57]. Alongside this, there is an elevation in fibrinogen levels, while the levels of prothrombin and platelets exhibit only minor alterations [58].

### 5.3. Association between Ocular Involvement and Disease Severity

In a retrospective study using the electronic medical records of 342 patients at a large tertiary academic medical center in India, the authors reported that 42.9% of patients with severe COVID-19 disease displayed ophthalmic manifestations as compared to 26.7% of patients with non-severe disease. This difference was found to be statistically significant (*p* value = 0.003). Loffredo et al. reported an overall rate of conjunctivitis in patients with a confirmed COVID-19 infection to be 3% and 0.7% in severe and non-severe illness. They further added that the presence of ocular manifestations may be a warning sign for poor outcomes. Similarly, Navel V et.al. in their study also reported that in individuals diagnosed with COVID-19, the presence of conjunctivitis typically correlates with a heightened severity of illness and a poorer prognosis.

## 6. Ocular Side Effects of COVID-19 Vaccines

### 6.1. Overview of COVID-19 Vaccine Types

Since the declaration of COVID-19 as a pandemic by the WHO on 11 March 2020, there have been 336 vaccine candidates developed, with 32 vaccines currently authorized for global use [59] (Table 2). Owing to the urgency of the pandemic and the rapid escalation of cases and fatalities, several of these vaccines proceeded directly to human clinical trials without prior pre-clinical testing on animals. Among them, 11 vaccine candidates received emergency use authorization (EUA) [60].

These vaccines can be categorized into four main groups: mRNA vaccines (such as BNT162b2 by Pfizer-BioNTech and mRNA-1273 by Moderna), vector vaccines (including Ad26.COV2 by Janssen Johnson & Johnson and ChAdOx1 nCoV-19/AZD1222 by Oxford-AstraZeneca), protein subunit vaccines (like NVX-CoV2373 by Novavax), and whole virus vaccines (such as PiCoVacc by Sinovac and BBIBP-CorV by Sinopharm) [60].

Among the mRNA vaccines, BioNTech and Pfizer developed two candidates, BNT162b1 and BNT162b2. Both are nucleoside modified, LNP encapsulated mRNA vaccine candidates [72]. mRNA-1273 is an LNP-encapsulated mRNA with an N1-methyl-pseudourine substituting uridine. It was developed by Moderna, and the mRNA encodes the SARS-CoV-2 full length S-2P protein [73]. ARCoV is another LNP encapsulated nucleoside modified mRNA vaccine. This vaccine candidate gave mice complete protection from a mouse-adapted SARS-CoV-2 strain in a preclinical study [61]. The Ad26.COV2 uses modified adenoviral DNA that encodes for a key part of the SARS-CoV-2 virus particle that our immune system can elicit a response against. This vaccine biochemistry is based on stable DNA molecules, so it does not require ultracold storage and is easy to store and distribute [74].

Since most of these vaccines received EUA, the data on the safety profile and potential side effects of these vaccines was limited. To manage this, the Centers for Disease Control and Prevention (CDC) expanded its Vaccine Adverse Event Reporting System (VAERS). VAERS is a national early warning system and functions as a passive surveillance platform for potential vaccine adverse events. The data collected by this platform is available through the Wide-Ranging Online Data for Epidemiologic Research platform (WANDER), which is also developed and operated by CDC [75].

In discussing the ocular side effects of COVID-19 vaccines, it is essential to maintain a balanced perspective. While reporting adverse events is critical for comprehensive understanding, it is equally important to emphasize their rarity relative to the widespread benefits of vaccination. Current data indicates that ocular complications post-vaccination, such as uveitis or retinal issues, are exceedingly rare, occurring in a small fraction of vaccinated individuals. These events must be contextualized within the broader context of millions of vaccine doses administered worldwide, where the overwhelming majority of recipients experience no significant ocular side effects. Emphasizing this rarity underscores the overall safety profile of COVID-19 vaccines while ensuring transparency in reporting potential adverse outcomes.

### 6.2. Reported Ocular Side Effects

The potential ocular side effects following COVID-19 vaccine administration in human are summarized in Table 3.

#### 6.2.1. Cornea

According to the VAERS database, 73% of the total reported cases of vaccine-associated corneal graft rejection were following vaccination with BNT162b2 vaccine and 26% were following the mRNA-1273 vaccine. The database analysis also reported that 61.87% of the rejections happened following penetrating keratoplasty (PKP), followed by 18.18% with Descemet stripping endothelial keratoplasty and 12.73% with Descemet membrane endothelial keratoplasty [101]. Corneal graft rejection after penetrating keratoplasty (PKP)—Wasser LM et al. reported the cases of two patients aged 56 and 73, with a history of penetrating keratoplasty presenting with acute corneal graft rejection 2 weeks after receiving the first dose of the BNT162b2 mRNA vaccine. Both patients were treated with hourly 0.1% dexamethasone and 60 mg per day of oral prednisone. The management resulted in prompt resolution of the graft rejection [77]. Rallis KI et al. described a case of a 68-year-old man who underwent penetrating keratoplasty presenting with acute corneal graft rejection three days after receiving the first dose of the BNT162b2 mRNA vaccine. The patient presented with discomfort in the eye, conjunctival hyperemia, and epithelial rejection line. Treatment with dexamethasone 0.1% eye drops hourly and oral acyclovir 400 mg five times daily for 1 week resulted in complete resolution of the symptoms [80]. Another study from the USA highlighted a similar presentation of a 51-year-old man with a history of PKP presenting with corneal graft edema and keratoprecipitates as signs of acute graft rejection 3 days after receiving the first dose of the mRNA-1273 vaccine. The patient was treated with topical steroid eye drops and this resulted in complete resolution of the graft rejection [102].

Corneal graft rejection after Descemet’s membrane endothelial keratoplasty (DMEK)—Phylactou M et al., in their study, reported the case of an 83-year-old woman who had undergone bilateral DMEK for Fuch’s endothelial corneal dystrophy and presented with acute endothelial graft rejection in both eyes 3 weeks after receiving the second dose of the BNT162b2 vaccine. She was treated successfully with topical steroids [82]. Crnej A et al. studied the case of a 71-year-old man who had undergone DMEK for endothelial decompensation post phacoemulsification and presented to the clinic with a sudden painless decrease in vision. On examination, he showed conjunctival injection and diffuse corneal edema, and a diagnosis of acute corneal edema was made. The patient was managed with topical steroids and oral valacyclovir resulting in symptom resolution in a week [81].

#### 6.2.2. Uvea

A retrospective observational study of the CDC-VAERS database reported 1094 cases of vaccine-associated uveitis from over 40 countries after SARS-CoV-2 vaccination. Among them, 77.97% of the cases were reported after the BNT162b2 vaccine, followed by 20.11% of the cases with the mRNA-1273 vaccine. A significantly higher number of cases were reported after the first dose of the vaccine and within 1 week of the administration of that dose [103].

Renisi et al. reported a case of a 23-year-old man who presented with a red eye associated with pain and photophobia two weeks after getting the second dose of the BNT162b2 vaccine. The patient had perichoretic and conjunctival hyperemia on examination. A diagnosis of acute anterior uveitis was made, and the patient was treated with topical steroids and cycloplegic drops [83]. Mudie et al. described a case of a 43-year-old woman who came to the clinic with the presentation of pan uveitis three days after receiving the second dose of the Pfizer-Biontech mRNA vaccine. The examination revealed vitreous inflammation and significant thickening of the choroid. The patient was treated with oral and topical steroids that resulted in resolution of the symptoms [87]. Goyal M et al. described a case of bilateral choroiditis following COVID-19 vaccination. The patient showed severe choroidal thickening and a large serous detachment of the macula on examination. There was a rapid improvement of the symptoms after initiation of oral steroids [28,104].

#### 6.2.3. Retina

Bøhler et al. described the case of a 27-year-old woman who presented to the clinic with visual disturbances a few days after receiving the first dose of the AstraZeneca vaccine. On examination, a paracentral scotoma was noted on perimetry in the upper temporal quadrant of the left eye. On fundoscopic examination, a tear-shaped macular lesion was noted, which was better visualized on optical coherence tomography. A diagnosis of acute macular neuro-retinopathy (A.M.N) was established [94]. Another study published a case of a 41-year-old woman who presented to the clinic with foggy vision 2 days after receiving the AstraZeneca vaccine. On fundoscopic examination, disc edema was noted in the right eye and a dome shaped serous detachment was noted in the upper quadrant of the left eye. A diagnosis of idiopathic optic disc edema was made for the right eye and that of central serous chorioretinopathy was made for the left eye [105].

Singh RB et al. analyzed cases of retinal vessel occlusion post COVID-19 vaccination from the VAERS database and reported that a majority (74.17%) of the cases of retinal vessel occlusion were reported after vaccination with the BNT162b2 vaccine, and 41.12% of the cases of retinal venous occlusion (RVO) and 48.27% of the cases of retinal arterial occlusion presented within the first week after vaccination [106]. Subramony R et al. reported the case of an otherwise healthy 22-year-old woman who presented to the emergency department with progressive painless loss of vision in her right eye a few days after receiving the Moderna SARS-CoV-2 vaccine. On examination, a point of care ultrasound showed bilateral retinal detachment, for which she subsequently underwent bilateral vitrectomies for management [26]. Based on the cases described, it is suggested that vaccine administration may have direct or indirect correlations with local ocular effects such as acute macular neuro-retinopathy, idiopathic optic disc edema, central serous chorioretinopathy, and retinal vessel occlusions, highlighting the need for further investigation into these associations.

#### 6.2.4. Other Reported Ocular Side Effects

Garcia-Estarada C et al. reported a case of a 19-year-old woman who developed optic neuritis 1 week after receiving a first dose of the Ad26.COV2.S vaccine [95]. Another study described a case of acute abducens nerve palsy in a 23-year-old man 1 week after receiving the Covishield vaccine [107]. Colella G et al. studied the case of a healthy 37-year-old who developed facial palsy within a few days after receiving the BNT162b2 mRNA vaccine [108]. Additional reports in the published literature have documented ocular manifestations following COVID-19 vaccination, including conditions such as thyroid eye disease, Miller Fisher syndrome, glaucoma, superior ophthalmic vein syndrome, and herpetic eye disease [109,110,111,112,113].

## 7. Mechanisms of Ocular Side Effects

Cunningham et al. attributed the immune responses observed in vaccine-associated uveitis (VAU) to a delayed type of hypersensitivity response to the molecular similarities between the vaccine peptides and the uveal self-peptides [114]. Rabinovitch et al. described a type 1 hypersensitivity reaction with the upregulation of proinflammatory molecules after interaction with the vaccine components [115]. The presence of autoimmune processes due to the upregulation of RNA sensing molecules like TLR3 and MDA5 in response to the mRNA molecules used in vaccine delivery was also noted [116]. There is evidence of similarities between the uveitic reaction observed post adenoviral infection, which was attributed to an antigen–antibody complex mediated type three hypersensitivity reaction and VAU in COVID-19 [117].

The complication of corneal graft rejection post vaccination is postulated to be due to cross reactivity between the human leukocyte antigens of the corneal endothelial cells and the viral antigen-specific T cells [83]. Immunologically, it has been observed that the proinflammatory state due to the body’s immune response to the SARS-CoV-2 vaccines leads to upregulation of the CD4 Th1 cells, and these cells have been shown to be one of the primary mediators of corneal graft rejection [82]. Furthermore, the interaction between the vaccine components and platelet factor 4 causing subsequent platelet activation has been postulated to be the underlying mechanism of the prothrombotic side effects of the SARS-CoV-2 vaccines like CRAO and CRVO [118,119].

## 8. Conclusions

In summary, ocular involvement in COVID-19 presents a complex challenge that warrants ongoing attention. From conjunctivitis to retinopathy and potential vaccine-related complications, thorough eye examinations and patient histories are crucial. Future research should focus on clarifying how SARS-CoV-2 affects different ocular tissues, understanding the long-term implications, and assessing emerging therapies. Vigilant surveillance and targeted studies will enhance our understanding and management of ocular manifestations in COVID-19, contributing to improved patient outcomes.

## Figures and Tables

**Table 1:** Ocular complications of an underlying SARS-CoV-2 disease.

Ocular Complication	Age and Sex	Ocular Presentation	Systemic Symptoms	Diagnostic Test for COVID-19	Symptom Timeline	Study
Conjunctivitis	41 M	Chemosis, epiphora, photophobia, eye lid edema, watery discharge, follicular reaction of the palpebral conjunctiva	None	RT-PCR		Scalini S et al. [21]
Anterior scleritis	67 F	Redness, pain, and photophobia in both eyes	Fever, headache, myalgia, dry cough, and dyspnea.	RT-PCR	Ocular symptoms presented 1 week after systemic symptoms	Feizi S et al. [22]
	33 M	Red eye, foreign body sensation, epiphora, and photophobia	Fever, myalgia, anosmia, ageusia, dry cough.	RT-PCR	Ocular symptoms 2 weeks after systemic symptoms	Feizi S et al. [22]
Episcleritis	29 M	Redness and foreign body sensation.	Headache cough, and fever.	RT-PCR	Ocular symptoms presented 3 days before systemic symptoms	Otiaf W et al. [23]
Central retinal vein occlusion	17 F	Diminished vision, optic disc examination—disc swelling, splinter hemorrhages, flame shaped and blot hemorrhages, OCT—neurosensory detachment and cystoid macular edema	Cough and fever.	RT-PCR	Ocular presentation occurred 21 days after the systemic presentation	Walinjkar et al. [24]
Bilateral central retinal vein occlusion	40 M	Blurring of vision in both eyes	Fever, cough, and shortness of breath.	RT-PCR	Systemic symptoms began 3 days before the ocular presentation	Gaba WH et al. [25]
Vasculitis RVO	52 M	Inferior hemiretinal vein occlusion with macular edema	Cough, fever, and malaise.	RT-PCR	Ocular symptoms presented 10 days after systemic ones	Sheth et al. [26]

**Table 2:** Vaccines that received Emergency Use Authorization (EUA).

Sno	Name	Vaccine Type	Route of Administration	Country of Development	Reported Efficacy against Mild COVID	Study
1	BNT163b2 Pfizer BioNTech	RNA (modified nucleoside in lipid nanoparticle)	Intra-muscular	Germany	95%	[61]
2	mRNA-1273 Moderna: Spikewax	RNA (modified nucleoside in lipid nanoparticle)	Intra-muscular	Spain (Moderna Biotech)	94.1%	[62]
3	Covovax (Novavax formulation)	Protein Subunit	Intra-muscular	India (Serum Institute of India)	89.7%	[63]
4	Nuvaxovid (Novavax)	Protein Subunit	Intra-muscular	Czech Republic	92.6%	[64]
5	Covishield (ChAdOx1 nCoV-19)	Non-replicating viral vector	Intra-muscular	India (Serum Institute of India)	90%	[65]
6	Jcovden: Janssen (Johnson & Johnson)	Non-replicating viral vector	Intra-muscular	Belgium	72%	[66]
7	Vaxzevria (Oxford AstraZeneca)	Non-replicating viral vector	Intra-muscular	Republic of Korea	70.4%	[67]
8	Convidecia: CanSino (Ad5.CoV2-5)	Non-replicating viral vector	Intra-muscular	People’s Republic of China	-	[68]
9	Sinopharm: Covilo/BBIBP-CorV	Inactivated	Intra-muscular	China (BIBP)	-	[69]
10	Sinovac: Coronavac	Inactivated	Intra-muscular	China (Sinovac Biotech)	67.7%	[70]
11	Covaxin	Inactivated (whole virion)	Intra-muscular	India (Bharat Biotech)	93.4%	[71]

**Table 3:** Ocular manifestations after vaccination against COVID-19 infection.

Ocular Side Effect	Age (years)/Age Range Study Type	Type of Vaccine	Symptoms	Time of Onset	Management	Study
Endothelial Graft Rejection	Median age 68 (27–83) IQR Systematic Review	BNT162b2 (mRNA) vaccine 8 patients	Hyperemia, diffuse conjunctival edema, flare, keratoprecipitates	17 days 7 days 3 days 14 days 9 days 13 days 3 weeks 4 days	Oral methyl prednisone, dexamethasone eye drops 0.2% hourly and hypertonic saline	Fujio K et al. [76]
		mRNA-1273 8 patients		1 week 1 week 2 weeks 1 week 15 days 3 days 1 week 1 week		
		ChAdOx1 4 patients		5 days 10 days 2 days 6 weeks		
		CoronaVac		1 day		
	73 yrs 56 yrs Case report	BNT162b2 (mRNA) vaccine	Discomfort in the eye, ciliary injection, corneal edema, Descemet folds and keratoprecipitates	2 weeks	Dexamethasone 0.1% eye drops hourly and oral prednisone 60 mg daily	Wesser LM et al. [77]
	94 yrs Case report	BNT162b2 (mRNA) vaccine	Painless worsening of vision	2 weeks	Topical dexamethasone and tobramycin	Forshaw, T et al. [78]
	71 yrs Case report	BNT162b2 (mRNA) vaccine	Conjunctival injection, sudden decrease in vision	1 week	Topical dexamethasone sodium 1 mg/mL/2 hourly	Crnej, A et al. [79]
	68 yrs Case report	BNT162b2 (mRNA) vaccine	Discomfort in the eye, conjunctival hyperemia, and epithelial rejection line.	1 day	Dexamethasone 0.1% eye drops hourly and acyclovir 400 mg 5 times a day for a week	Rallis et al. [80]
	Case series	BNT162b2 (mRNA) vaccine 3 cases	Decreased visual acuity, ocular pain, photophobia	16.86 ± 6.96 days (mean)	Topical steroids	Molero-Senosiain, et al. [81]
		AZD1222 2 cases		17 ± 11.89 days		
	83 yrs 66 yrs Case report	BNT162b2 (mRNA) vaccine	Discomfort, painless decrease in vision hyperemia	7 days 3 weeks	Topical steroids	Phylactou et al. [82]
Acute Anterior Uveitis	23 yrs Case report	BNT162b2 (mRNA) vaccine	Pain, photophobia and conjunctival hyperemia	14 days	Topical steroids and cycloplegic eye drops for 10 days	Renisi et al. [83]
	46 yrs Case report	BNT162b2 (mRNA) vaccine	Photophobia, pain, blurring of vision	3 weeks	Azathioprine 50 mg once daily Topical triamcinolone drops	Al-Allaf, et al. [84]
	92 yrs 85 yrs Case Report	BNT162b2 (mRNA) vaccine	Hyperemia, ocular pain, headache	3 days 3 days	Cycloplegic eye drops every 8 h and moxifloxacin every 4 h	Ortiz Egea et al. [85]
	79 yrs 55 yrs Case report	BNT162b2 (mRNA) vaccine	Keratoprecipitates, vitreous opacity, inflammatory cells in the anterior chamber	3 days 2 days	Topical steroids	Choi et al. [86]
	62 yrs Case report	AZD1222		1 day		
Panuveitis	43 F	BNT162b2 (mRNA) vaccine	On examination—vitreous inflammation and thickening of the choroid	2 weeks	Oral and topical steroids	Mudie et al. [87]
Herpes Zoster Ophthalmicus	29 yrs 34 yrs Case report	BNT162b2 (mRNA) vaccine	Painful grouped vesicles in the S3 dermatome, painful inguinal lymph nodes	15 days 13 days	Valacyclovir 1 g 3×/day for 10 days	Van Dam, C. S et al. [88]
	58 yrs 47 yrs 39 yrs 56 yrs 41 yrs Case series	BNT162b2 (mRNA) vaccine	Umbilicated vesicles, rash in dermatomal pattern, lymphadenopathy	1 day 5 days 3 days 2 days 16 days	Valacyclovir 1 g 3×/day for 7 days	Rodriguez-Jimenez P et al. [89]
	68 yrs Case report	Inactivated COVID-19 vaccine	Erythematous rash with vesicular lesions, stinging sensation and pain	5 days	Valacyclovir 1 g 3×/day for 7 days	Aksu, S.B et al. [90]
	51 yrs 56 yrs 89 yrs 86 yrs 90 yrs 91 yrs 94 yrs Case Series	BNT162b2 (mRNA) vaccine	Dermatomal rash, vesicles, pain, headache	9 days 2 weeks 8 days 1 week 9 days 1 week 10 days	Acyclovir 5×/day, prednisone phosphate 0.5%, doxycycline 50 mg orally once a day	Psichogiou M, et al. [91]
Acute macular neuro retinopathy	27 yrs 22 yrs 28 yrs Case report	AZD1222	Paracentral scotoma with a macular lesion nasal to the fovea	2 days 2 days 2 days	Self-limiting	Mambretti, et al. [92]
	21 yrs	AZD1222	Bilateral paracentral scotoma, OCT with outer plexiform layer thickening	3 days	Self-limiting	Book, et al. [93]
	27 yrs Case report	AstraZeneca	Visual disturbances, on examination—paracentral scotoma, tear shaped macular lesion		-	Bøhler et al. [94]
Retinal detachment	22 yrs Case report	mRNA-1273 (Moderna)	Progressive painless loss of vision		Vitrectomy	Subramony R et al. [26]
Optic Neuritis	19 yrs Case report	Ad26.COV2.S	Eye pain, pain with movement of the eye		Oral steroids	Garcia-Estarada C et al. [95]
Retinal vein occlusion	50 yrs 56 yrs Case report	BNT162b2 (mRNA) vaccine	Sudden onset vision loss, reduced best corrected visual acuity (BCVA) on examination	3 days 3 days	Intravitreal ranibizumab	Tanaka H, et al. [96]
	58 yrs 73 yrs	AstraZeneca	Pianless decrease in vision	3 days 3 days	Intravitreal ranibizumab	Peters MC, et al. [97]
	47 yrs 36 yrs Case series	BNT162b2 (mRNA) vaccine		5 days 1–3 days		
	50 yrs 43 yrs Case report	AstraZeneca	Sudden onset blurry vision	4 days 3 days	Intravitreal ranibizumab Close follow up	Sonawane NJ, et al. [98]
	66 yrs 51 yrs 66 yrs 54 yrs Case series	AstraZeneca	Painless vision loss, cotton wool spots on examination	16 days 6 days 4 days 10 days	Intravitreal steroids	Da Silva LSC, et al. [99]
	71 yrs 72 yrs	BNT162b2 (mRNA) vaccine	Blurry vision, decreased BCVA	1 day 1 day	Intravitreal ranibizumab	Tanaka H, et al. [100]
